# The Influence of Online Game Behaviors on the Emotional State and Executive Function of College Students in China

**DOI:** 10.3389/fpsyt.2021.713364

**Published:** 2021-10-20

**Authors:** Wei Zhao, Tao Wei, Ruidong Zhou, Yujing Wang, Yan Wang, Zixuan Ren, Wenyi Shao, Hanrun Luo, Yiding Zhou, Nuo Chen, Qiao Lu, Xun Song, Ziyao Zhang, Yinnuo Fang, Xinyi Zhang, Dongliang Jiao

**Affiliations:** School of Mental Health, Bengbu Medical College, Bengbu, China

**Keywords:** Internet Game Addiction, executive function, anxiety, depression, college students

## Abstract

**Background and Objective:** Since the classification of gaming disorder (GD) by the World Health Organization (WHO) as “mental disorder caused by addictive behaviors,” there has been controversy regarding whether online game behaviors can lead to mental disorder. This study aims to clarify the correlation between the online game behaviors of college students and anxiety, depression, and executive function of college students in China, from a questionnaire-based investigation.

**Methods:** Based on the whole class random sampling method, a questionnaire survey was conducted among college students in Northern Anhui, China from March 7 to March 27, 2020. The questionnaires included the Internet Game Addiction (IGA) Scale, Behavior Rating Inventory of Executive Function (Adult Version, BRIEF-A), Generalized Anxiety Disorder Scale (GAD-7) and Patient Health Questionnaire Scale (PHQ-9).

**Results:** A total of 850 participants completed the survey, including 353 males (41.53%) and 497 females (58.47%). The primary age group was 18–27 years (91.53%), and the educational background was a bachelor's degree (94.7%). The study found that the online behavior of 17.76% of college students was online game behavior. This study did not identify any students who met the criteria for IGA, and 3% met the criteria for indulgent behavior. A dual role of online games was identified; moderate online game activities can improve the emotional state and executive function of college students, while excessive online game behaviors that may not reach the degree of addiction can also harm emotional state and executive function.

**Conclusions:** This study suggests that although IGA has been regarded as a mental disease, online game behavior should be treated differently. Online game activities should not be entirely denied, but mental disorders caused by excessive gaming activities deserve attention. In particular, the emotional state and executive function of students with excessive online game behaviors should be monitored and intervened in advance to avoid game behaviors turning into indulgent behaviors or addiction. As a cognitive control process, executive function may play a key role in regulating IGA and emotional state.

## Introduction

With the rapid growth of the online game market, many young men indulge in online games, which has resulted in lots of negative social effects. Problems related to IGA have become increasingly concerning. Previous research reported that the prevalence of IGA has been estimated to be 0.5–6% (1, 2). Long et al. (3) analyzed 36 representative investigations and found that the prevalence of problematic IGA was 3.5–17%. King et al. investigated more than 3,000 subjects and found that the prevalence of IGA was 0.3–4.9% (4). In addition, the above studies have found that young people are the high incidence population of IGA. The individual susceptibility constituted by genetic, physio genesis and personality characteristics may predispose young people to addictive tendencies and indicates that IGA disorder of young people is a real problem that needs attention.

IGA has been considered as a clinical phenomenon that requires further study, per the Diagnostic and Statistical Manual of Mental Disorders, 5th Edition, 2013 (DSM-5) (5). In 2019, the World Health Organization (WHO) regarded gaming disorder as a mental disease and classified it in the category of the disorders caused by addictive behaviors (6). The introduction of two new standards has guided the primary direction for research in this field (7), but has also generated significant controversy. Clinical studies have found that IGA has the characteristic similar to those of behavioral addiction, such as excessive attention-seeking, compulsive, lack of control and impulsive behavior (8). Related research has indicated that IGA may decrease sleep quality and has a strong correlation with different degrees of anxiety, depression and other psychological distress (9, 10). However, some researchers have suggested that IGA should not be classified as a mental disease (11). It has been reported that online games bring happiness to players, reduce anxiety, depression (12) and improve cognitive function (13). In addition, most online gamers are not exposed to addiction, suggesting the presence of factors mitigating against IGA. Previous studies highlighted that executive function plays a key role in the regulation of addictive behaviors and emotional status (14). Executive function includes decision-making, planning, inhibition and behavioral shift (15). Moreover, executive function can prevent the development of addiction by inhibiting impulsive and controlling behaviors, whilst degradation of executive function leads to an increase in impulsive behaviors and addiction development (16). Meanwhile, excessive online gaming could be detrimental to executive function, consequently spiraling without control and leading to impulsive behaviors (17). In essence, executive function plays a key role in IGA mitigation and mental health.

This study focused on the roles of executive function, anxiety and depression on the development of IGA, based on the Behavior Rating Inventory of Executive Function (Adult Version, BRIEF-A), Generalized Anxiety Disorder Scale (GAD-7) and Patient Health Questionnaire Scale (PHQ-9). Since online gamer demographics are predominantly individuals in the younger age-bracket, college students were selected for becoming participants of this study. This investigation sheds further light on the correlation of IGA and emotional disorder with executive function disorder, thus providing references to IGA mitigation within clinical practice.

## Method

### Research Subjects

Based on the whole class random sampling method, a questionnaire survey was conducted on college students in Northern Anhui Province, China from March 7 to March 27, 2020. An online questionnaire survey method was used for the study and more than two classes were randomly selected from each grade of each school. With the help of school counselors, questionnaire links were distributed in a QQ group or WeChat group, and only one questionnaire could be completed per IP address. Questionnaires that were completed too quickly were eliminated and 850 valid questionnaires were finally considered for the study. Bengbu Medical College Institutional Review Board authorized this study (approval number: 2019-199). All experiments were performed in compliance with the regulatory approval.

### Research Tools

#### IGA Scale

Currently, there are few measurement tools for the diagnosis and evaluation of IGA which are based on DSM-5 and ICD-11. However, there are several inconsistencies between the two criteria used for determining the prevalence of IGA (7), which in turn, leads to a significant difference in determining the incidence of IGA. The development of measurement tools needs to be validated in cross-cultural clinical samples for effectiveness and reliability. Better results would be obtained if objective indicators including autonomic nervous system response, and electrophysiological parameters can be measured (5, 18, 19). Based on the above reasons, the IGA scale was used in this study. The IGA scale was compiled by Chinese scholars, which used Chinese college students as samples and adopted an event-related brain potential method to identify objective electrophysiological indexes that could distinguish IGA users from other Internet users. The reliability and stability of the scale for Chinese young people have been proven by reliability and validity tests (20, 21).

IGA scale with 11 questions and a Likert 5-point scoring method were used for the study. From being “highly consistent” to “very inconsistent,” the consistency between the actual situation of the subjects and the questionnaire items were correspondingly scored from “4 points” to “0 points,” respectively. Subjects with higher scores were more likely to develop IGA. A score < 20 was regarded as normal online game behavior, a score ≥ 20 but <30 was regarded as online game indulgent and a score ≥ 30 was considered as IGA. In this study, the Internal consistency Cronbach's α of the questionnaire was 0.856, and the KMO test coefficient (Bartlett's test, *P* < 0.05) was 0.828, indicating that the scale had good reliability and validity.

#### The Generalized Anxiety Disorder Scale

The Generalized Anxiety Disorder Scale (GAD-7) was previously translated into Chinese and validated by researchers in China (22). GAD-7 is a quantitative evaluation standard recommended by DSM-5 published by the American Psychiatric Association. It is an effective tool to identify possible cases with generalized anxiety disorder and has shown good reliability and validity in previous studies. The score is divided into four levels: 0–4, 5–9, 10–14, and 15–21, corresponding to no, mild, moderate and severe anxiety, respectively (23). In this study, the Cronbach's α of the standardized item of the scale was 0.922, and the KMO test coefficient (Bartlett's test, *P* < 0.05) was 0.920, indicating that the scale had good reliability and validity.

#### Patient Health Questionnaire Scale

The Patient Health Questionnaire Scale (PHQ-9) was previously translated into Chinese and validated by researchers in China (24, 25). PHQ-9 is based on nine criteria of depression as stated in DSM-5 and is highly sensitive to depressive symptoms. The score is divided into five levels: 0–4, 5–9, 10–14, 15–19, and 20–27 corresponding to no, mild, moderate, moderately-severe and severe anxiety, respectively (26). In this study, the Cronbach's α of the standardized item of the scale was 0.905, and the KMO test coefficient (Bartlett's test, *P* < 0.05) was 0.930, indicating that the scale had good reliability and validity.

#### Executive Function Scale

Executive function was measured with the Chinese version Behavior Rating Inventory of Executive Function-Adult Version (BRIEF-A) developed by Roth and Gioia (27), It contains 75-items that yield an overall score, the Global Executive Composite(GEC), which is derived from two index scores [Behavioral Regulation Index (BRI) and Metacognitive Index (MI)]. The BRI is comprised of four clinical scales: Inhibit, Shift, Emotional Control and Self-Monitor. The MI is comprised of five clinical scales: Initiate, Working Memory, Plan or Organize, Task Monitor and Organization of Materials. A 1–3 level scoring system was adopted, with a score of 1 for “never,” a score of 2 for “sometimes” and a score of 3 for “often.” The higher the total score, the more serious the impairment of executive function. In this study, the internal consistency Cronbach's α of this scale was 0.976, and the KMO test coefficient (Bartlett's test, *P* < 0.05) was 0.966, indicating that the scale had good reliability and validity.

### Statistical Analysis

The SPSS 25.0 software was used for statistical analysis in this study. The measured data were expressed as (M ± SD), and the independent sample *t*-test was used to compare two groups of measured data. The measured data for multiple groups were analyzed by one-way ANOVA, and multiple comparisons were made. Also, correlation analyses and multiple linear regressions were used to identify the relationships between the IGA score and anxiety, depression, and executive function, with a test level α of 0.05.

## Results

### General Demographic Data of Subjects

A total of 850 participants completed the survey, including 353 males (41.53%) and 497 females (58.47%). The overall age distribution was between 18 and 27 years (91.53%). The educational background of the subjects mostly comprised a bachelor's degree (94.7%). Other general demographic data are shown in Table 1.

**Table 1:** General demographic data of subjects (*n* = 850).

	**Variables**	**Number**	**Percentage (%)**
Total		850	100
Gender	Male	353	41.53
	Female	497	58.47
Age	<18	34	4.00
	18–23	677	79.65
	24–27	101	11.88
	>27	38	4.47
Grade	Freshman	200	23.53
	Sophomore	98	11.53
	Sophomore	287	33.76
	Junior	180	21.18
	Senior	40	4.71
	Postgraduate or above	45	5.30
Residence	Country	363	42.71
	Town	195	22.94
	City	292	34.35

### Network Usage of Subjects

#### Primary Internet Behaviors of Subjects Over the Past 12-Month Period

**Figure 1:**
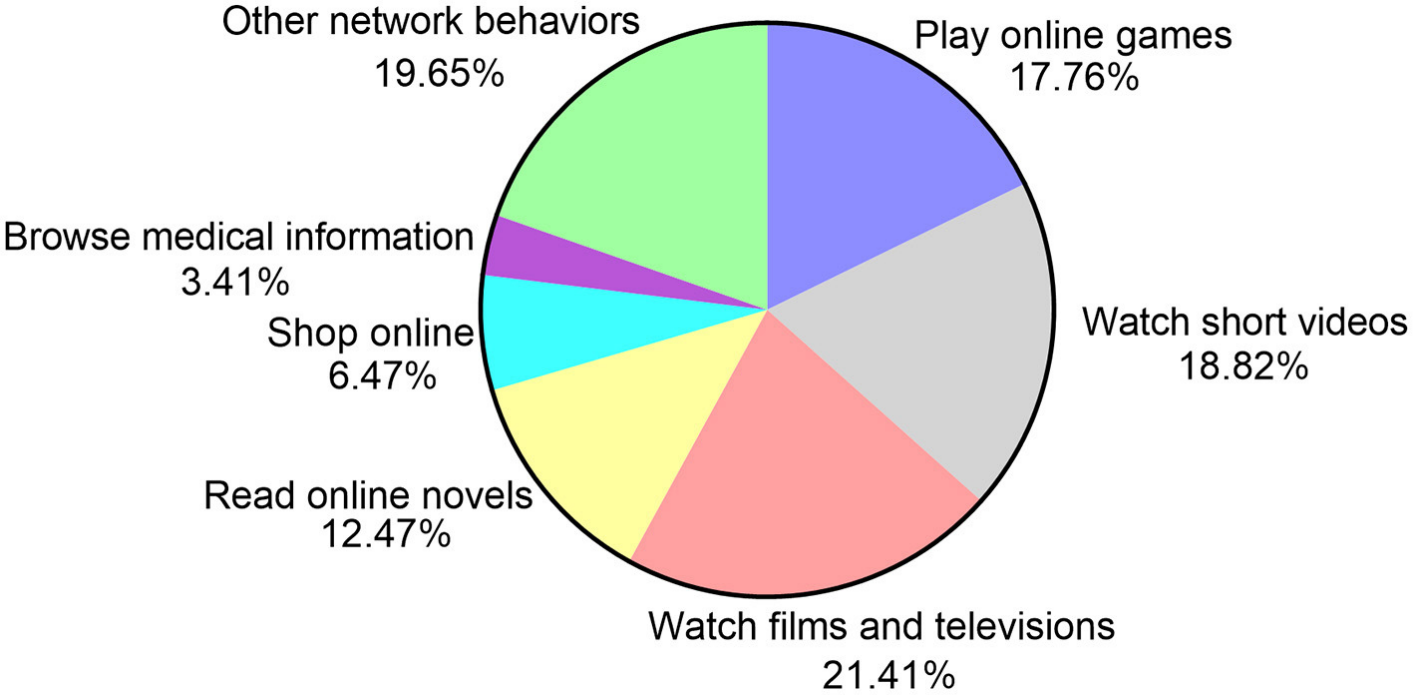
The primary network behaviors and grouping of participants (*n* = 850) over the past 12 months.

The 850 participants of the study were divided into two groups according to their primary Internet behaviors over the past 12 months. There were 151 (17.76%) participants in the Game group and the primary Internet behavior was playing online games. There were 699 (82.24%) participants in the Non-game group and the primary Internet behaviors included watching cinematographic and television programs, short videos, reading online novels, and shopping (Figure 1).

#### Basic Information of the Game and Non-game Groups

There were significant differences in gender, grade, and online time between the Game and Non-game groups (Table 2).

**Table 2:** Basic information of the game group and the Non-game group.

	**Variables**	**Network usage type**		**χ^2^**	***P***
		**Non-game(*n* = 699)**	**Game** **(*n* = 151)**		
Gender	Male	254 (36.3)	99 (65.6)	43.678	0.000^***^
	Female	445 (63.7)	52 (34.4)		
Age	<18	25 (3.6)	9 (6.0)	2.122	0.548
	18–23	557 (79.7)	120 (79.5)		
	24–27	85 (12.2)	16 (10.6)		
	>27	32 (4.6)	6 (4.0)		
Grade	Freshman	146 (20.9)	54 (35.8)	15.774	0.008^**^
	Sophomore	84 (12.0)	14 (9.3)		
	Sophomore	245 (35.1)	42 (27.8)		
	Junior	154 (22.0)	26 (17.2)		
	Senior	33 (4.7)	7 (4.6)		
	Postgraduate or above	37 (5.3)	8 (5.3)		
Residence	Country	298 (42.6)	65 (43.0)	1.822	0.402
	Town	155 (22.2)	40 (26.5)		
	City	246 (35.2)	46 (30.5)		
Online time	<1 h	27 (3.9)	17 (11.3)	17.305	0.002^**^
	1–2 h	77 (11.0)	13 (8.6)		
	2–4 h	199 (28.5)	33 (21.9)		
	4–8 h	265 (37.9)	53 (35.1)		
	>8 h	131 (18.7)	35 (23.2)		

** *P < 0.01*,

*** *P < 0.001*.

### Relationships Between IGA Scores and Anxiety, Depression, and Executive Function Scores

#### Differences in Anxiety and Depression Levels Among Groups

**Figure 2:**
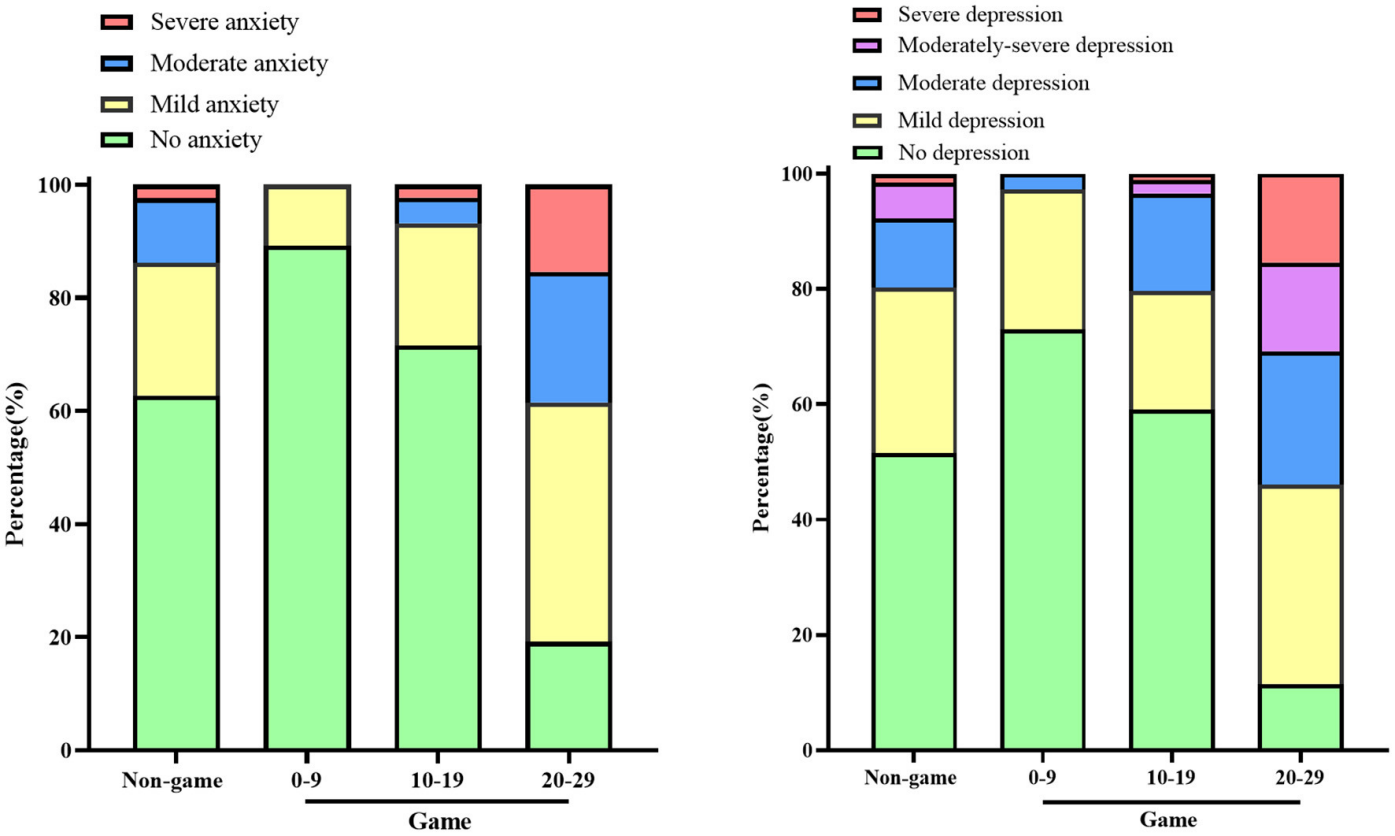
Different anxiety levels (GAD-7) and depression levels (PHQ-9) among groups without depression or anxiety accounted for the most significant proportion in the 0-9 score group, and subjects with more than moderate anxiety or depression accounted for the most significant proportion in the 20–29 score group. 0–9, 10–19, 20–29, respectively represents group whose scores of Internet Game Addiction Scale were 0–9, 10–19, 20–29.

Based on the IGA score, the Game group was further divided into a 0–9 score group (6.68 ± 2.16), a 10–19 score group (14.84 ± 2.24), and a 20–29 score group (22.65 ± 1.98). The results showed that subjects without depression or anxiety accounted for the most significant proportion of the 0–9 score group, and subjects with more than moderate anxiety or depression accounted for the most significant proportion of the 20–29 score group (Figure 2).

#### Scores of Anxiety and Depression, the Executive Function Total Scores, and the Executive Function Score of Each Factor Among Groups

**Figure 3:**
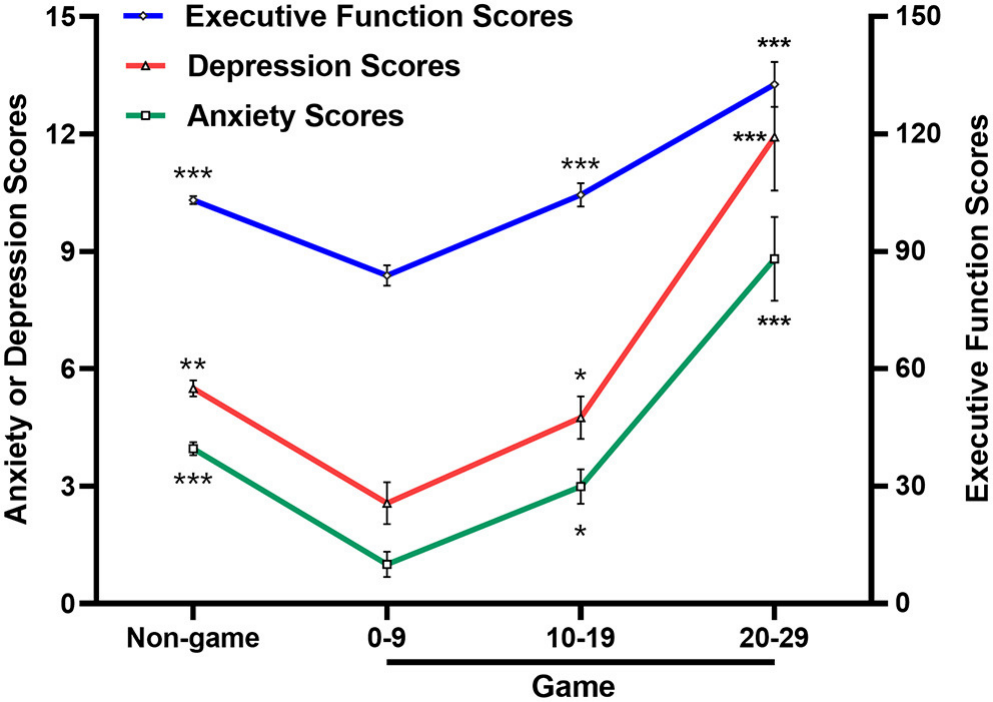
Trends of anxiety, depression, and total scores of executive function among groups. 0–9, 10–19, 20–29, respectively represents group whose scores of Internet Game Addiction Scale were 0–9, 10–19, 20–29; *P < 0.05: vs. 0–9 Game group, **P < 0.01: vs. 0–9 Game group, ***P < 0.001: vs. 0–9 Game group.

The results showed that students in the 0–9 score group (based on the IGA score) had lower scores on the anxiety, depression, and executive function scales than those in the Non-game group, 10–19 score group, and 20–29 score group (*P* < 0.05). However, students who were in the 20–29 score group had higher scores on the anxiety, depression, and executive function scales than those in other groups (*P* < 0.05) (see Table 3). These trends are shown in Figure 3.

**Table 3:** Scores of anxiety and depression, total scores, and the executive function scores of each factor among groups (M ± SD).

	**Non-game^a^** **(*n* = 699)**	**Game**			***F***	***P***	***Post-hoc***
		**0–9^b^** **(*n* = 37)**	**10–19^c^** **(*n* = 88)**	**20–29^d^** **(*n* = 26)**			
GAD-7	3.96 ± 4.49	1.00 ± 1.96	2.99 ± 4.11	8.81 ± 5.46	17.382	0.000^***^	d>c=a>b
PHQ-9	5.50 ± 5.32	2.57 ± 3.29	4.75 ± 5.13	11.92 ± 7.00	17.156	0.000^***^	d>c=a>b
BRIEF-A	103.07 ± 28.42	83.86 ± 15.68	104.42 ± 28.35	132.69 ± 29.23	15.544	0.000^***^	d>c=a>b
BRI	43.44 ± 12.66	35.35 ± 5.92	43.47 ± 12.30	57.08 ± 13.14	15.659	0.000^***^	d>c=a>b
Inhibit	11.61 ± 3.40	9.62 ± 1.75	11.65 ± 3.26	15.15 ± 3.04	14.293	0.000^***^	d>c=a>b
Shift	8.73 ± 2.80	6.95 ± 1.54	8.83 ± 2.64	11.38 ± 2.83	13.399	0.000^***^	d>c=a>b
Emotional control	14.50 ± 4.58	11.46 ± 2.12	14.22 ± 4.56	19.42 ± 5.88	15.810	0.000^***^	d>c=a>b
Self-monitor	8.60 ± 2.79	7.32 ± 1.47	8.77 ± 2.71	11.12 ± 2.66	10.071	0.000^***^	d>c=a>b
MI	59.63 ± 16.30	48.51 ± 10.28	60.95 ± 16.86	75.62 ± 17.10	14.474	0.000^***^	d>c=a>b
Initiate	12.51 ± 3.58	10.11 ± 2.00	12.95 ± 3.43	15.27 ± 3.55	11.582	0.000^***^	d>c=a>b
Working memory	11.90 ± 3.51	9.57 ± 2.27	11.94 ± 3.66	15.58 ± 3.11	15.292	0.000^***^	d>c=a>b
Plan/organize	14.69 ± 4.44	12.03 ± 3.31	14.94 ± 4.61	19.15 ± 4.70	13.357	0.000^***^	d>c=a>b
Task monitor	9.02 ± 2.66	7.30 ± 1.35	9.35 ± 2.64	11.23 ± 3.04	11.906	0.000^***^	d>c=a>b
Organization of materials	11.51 ± 3.42	9.51 ± 2.29	11.76 ± 3.71	14.38 ± 4.54	10.261	0.000^***^	d>c=a>b

*** *P < 0.001; GAD-7, The Generalized Anxiety Disorder Scale; PHQ-9, Patient Health Questionnaire Scale; BRIEF-A, Executive Function-Adult Version; BRI, Behavioral Regulation Index; MI, Metacognitive Index*.

#### Correlation Analysis of IGA Scores With Gender, Age, Anxiety, Depression, and Executive Function

Table 4 shows the correlation between IGA scores and gender, age, anxiety, depression, and executive function. The results showed that IGA scores were positively correlated with age, anxiety, depression, executive function, and executive function subscale factors (see Table 4).

**Table 4:** Correlation analysis between online game scale scores and gender, age, anxiety, depression, and executive function (*n* = 151).

	**IGA scale**	
	***r***	***P***
Gender	−0.004	0.960
Age	0.176	0.030^*^
Online time	0.143	0.079
GAD-7	0.556	0.000^***^
PHQ-9	0.533	0.000^***^
BRIEF-A	0.570	0.000^***^
BRI	0.575	0.000^***^
Inhibit	0.551	0.000^***^
Shift	0.559	0.000^***^
Emotional control	0.544	0.000^***^
Self-monitor	0.503	0.000^***^
MI	0.543	0.000^***^
Initiate	0.523	0.000^***^
Working memory	0.545	0.000^***^
Plan/organize	0.513	0.000^***^
Task monitor	0.528	0.000^***^
Organization of materials	0.449	0.000^***^

* *P < 0.05*,

*** *P < 0.001; IGA Scale, Internet Game Addiction Scale; GAD-7, The Generalized Anxiety Disorder Scale; PHQ-9, Patient Health Questionnaire Scale; BRIEF-A, Executive Function-Adult Version; BRI, Behavioral Regulation Index; MI, Metacognitive Index*.

#### Multiple Linear Regression Analysis of IGA Scores With Age, Anxiety, Depression, and the Total Score of Executive Function (*n* = 151)

The IGA score was treated as the dependent variable, and age, anxiety, depression, and executive function scores were treated as independent variables for multiple linear regression analysis. The Variance Inflation Factor (VIF) for the predictor variables ranged from 1.027 to 4.768, which is acceptable as being below the threshold of 10; likewise, Tolerance levels for each predictor ranged from 0.210 to 0.974, which is also a satisfactory range. The results indicated that age, anxiety, and the total score of executive function were independent influencing factors of online game behavior (see Table 5).

**Table 5:** Multivariate regression analysis (*n* = 151).

	**B**	**SE**	**Beta**	***t***	***P***
(Constant)	0.726	2.234		0.325	0.746
Age	2.139	0.641	0.213	3.336	0.001^**^
GAD-7	0.385	0.148	0.324	2.601	0.01^*^
PHQ-9	−0.053	0.129	−0.06	−0.413	0.68
BRIEF-A	0.075	0.018	0.406	4.151	0.000^***^

* *P < 0.05*,

** *P < 0.01*,

*** *P < 0.001, R^2^ = 0.419, Adjusted R^2^ = 0.403; GAD-7, The Generalized Anxiety Disorder Scale; PHQ-9, Patient Health Questionnaire Scale; BRIEF-A, Executive Function-Adult Version*.

## Discussion

This study investigated the differences in anxiety, depression, and executive function scores among the Non-game group and the Game group (the Game group was divided into the 0–9, 10–19, 20–29 score groups according to the IGA scale score). The results showed that the scores for anxiety, depression, and executive function of subjects in the 0–9 score group were lower than those in the Non-game group, 10–19 score group, and 20–29 score group. It indicated that compared with other online entertainment behaviors, moderate online games might help alleviate anxiety and depression, and improve executive function. This confirms prior research findings that moderate online games can improve players' cognitive executive ability (28, 29) and alleviate or even reduce players' anxiety, depression, and other negative emotions (30, 31). A brain electrophysiology study on games also showed that moderate games activate brain areas related to emotion processing and that the frontal lobe areas related to attention are also more activated (32).

However, this study found that the scores for anxiety, depression, and executive function of subjects with higher online game scores (20–29 scores) were significantly higher than those in the 0–9, and 10–19 score groups, and in the Non-game group. Further correlation and regression analyses for the Game group revealed that anxiety, depression, and executive function were significantly positively correlated with the IGA score. The above conclusions showed that although excessive online game behavior (20–29 scores) did not reach the degree of addiction (online game scores were 30 and above), it still had a certain degree of negative impact on the emotions and executive functions of players.

This study showed the correlation of emotion and addiction with executive function. As a key subcortical brain area (e.g., nucleus accumbent, amygdala, cingulum gyrus, hypothalamus), the limbic system is closely related to addiction development (33). Appropriate online gaming leads to increased release of dopamine within the limbic system, resulting in pleasure. Consequently, such gamers feel calm, with anxiety and depression being mitigated (34). Therefore, moderate gaming can maintain emotional volatility in balance, while IGA causes emotional imbalance. In order to pursue pleasure through online games, gaming addicts are unable to extricate themselves from playing, resulting in impulsive and uncontrollable behaviors. Abrupt gaming halts trigger negative emotions such as anxiety, depression and anger (35). At this stage, the purpose of online gaming addicts to compulsively play is not only a method for pleasure-seeking, also a means to offset negative emotions, such as anxiety and depression (36). This also describes why this study found that online gaming abusers have more serious anxiety and depression behavioral traits. Previous studies have found that IGA was significantly associated with anxiety, depression, and alexithymia (37).

Being one of the functions of the frontal cortex (15), executive function is also involved in addiction regulation (38). Indeed, executive function does include advanced brain functions, including planning, inhibiting, control, shift and decision-making (39). Executive function mitigates addiction by inhibiting impulse behaviors; normal executive function can maintain online gamer behavior in a moderate state and abate/stabilize emotional volatility. Impaired executive function leads to limbic system dysregulation, thus aggravating impulsive behavior and causing IGA (40). In addition, IGA can consequently aggravate executive function damage. For instance, executive function disorder is observed in online gaming addicts (41) and Studies of brain potentials (42) and functional magnetic resonance (43) have found that IGA causes executive dysfunction through frontal lobe injury.

This is consistent with the conclusion that gaming abusers are exposed to poor executive function. Hence, executive function may play a key role in the mitigation of IGA and regulation of emotional status.

It has been found that age has an inverted U-shaped relationship with GD, the risk peaks in puberty and decreases at ~30 years of age (44). In fact, while the limbic system undergoes remarkable remodeling during puberty, prefrontal areas development is not complete until near the age of 25 (45). In this study, though 96% of the participants in this study were over 18 years old, the number of people aged 18–25 accounts for nearly 80% of the total population. According to the neurobiological model of addiction, neurodevelopmental changes occurring during young people lead to an imbalance between emotional (reward motivation) and executive control (46, 47). This neurobiological fragility may contribute in young people to a higher risk of developing addictive behaviors (48).

This study also found that in the online game group, the proportion of males was significantly higher than that of females (*P* < 0.001), which is consistent with previous research (49, 50). The differences in user needs between males and females could be the possible reason. Researchers have indicated that males prefer to get novel, stimulating, and exciting game experiences through online games, while females tend to maintain a real relationship through online social chat (51).

According to the standards of the original scale, those with scores of 30 and above are considered to be IGA subjects. However, there were no subjects with scores of 30 and above in this study. The reason for this might be that the subjects selected focus only on college students. Well-educated college students have better self-management abilities and hence lower rates of addiction. Some studies have found that even among the general population, only 0.3–1.0% of people meet the diagnostic criteria of IGA (11). On the other hand, the sample size of this study was not adequately large.

## Limitation

We investigated college students in Northern Anhui, China, the sample size of this study was not adequately large and the research subjects were only college students. Therefore, the results of this study need to be verified with a larger sample size and an expanded scope of research. This study only used questionnaire survey, and the results might be partially subject to subjectivity. Further research that includes brain imaging and neurophysiology is also necessary to corroborate the results of this study.

## Conclusions

This study identified a dual role of online games. Moderate online game activities could improve the emotional state and executive function of college students. However, excessive online game behavior that does not reach the degree of addiction can also negatively affect emotional state and executive function. The imbalance between reward motivation and executive control might contribute to IGA. This study suggests that online game activities should not be completely denied, but the emotional state and executive function of those who indulge in online games should be monitored. Pre-intervention can prevent game behavior turning into an addiction.

## Data Availability Statement

The original contributions presented in the study are included in the article/supplementary material, further inquiries can be directed to the corresponding author.

## Ethics Statement

The studies involving human participants were reviewed and approved by Ethics Committee of Bengbu Medical College. Written informed consent to participate in this study was provided by the participants' legal guardian/next of kin. Written informed consent was obtained from the individual(s), and minor(s)' legal guardian/next of kin, for the publication of any potentially identifiable images or data included in this article.

## Author Contributions

WZ and TW wrote the first draft of the manuscript. DJ and RZ provided critical revision of the manuscript for important intellectual content. All authors have materially participated in the manuscript preparation and gave input to the manuscript text and approved the final version of the manuscript.

## Funding

Project supported by the provincial Natural Science Foundation of Anhui (1908085MH278), Shanghai Key Laboratory of Psychotic Disorders Open Grant (13dz2260500), Innovative training Program for Chinese College students (11910510067), Innovative training Program for Chinese Graduate students (Byycx20008), Bengbu City - Bengbu Medical College Joint Science and Technology Project (BYLK201822), Bengbu Medical College key Laboratory of Addiction Medicine, Innovative training Program for Chinese Graduate students (Byycx21025), Science and Technology Development Fund of Bengbu Medical College (BYKF1818), and Key projects of Natural Science in Bengbu Medical College (2020byzd022).

## Conflict of Interest

The authors declare that the research was conducted in the absence of any commercial or financial relationships that could be construed as a potential conflict of interest.

## Publisher's Note

All claims expressed in this article are solely those of the authors and do not necessarily represent those of their affiliated organizations, or those of the publisher, the editors and the reviewers. Any product that may be evaluated in this article, or claim that may be made by its manufacturer, is not guaranteed or endorsed by the publisher.
